# Polyvinyl Alcohol/Chitosan Single-Layered and Polyvinyl Alcohol/Chitosan/Eudragit RL100 Multi-layered Electrospun Nanofibers as an Ocular Matrix for the Controlled Release of Ofloxacin: an *In Vitro* and *In Vivo* Evaluation

**DOI:** 10.1208/s12249-021-02051-5

**Published:** 2021-06-03

**Authors:** Shahla Mirzaeei, Shiva Taghe, Kofi Asare-Addo, Ali Nokhodchi

**Affiliations:** 1 Pharmaceutical Sciences Research Center, Health Institute, Kermanshah University of Medical Sciences, Kermanshah, Iran; 2 Nano Drug Delivery Research Center, Health Technology Institute, Kermanshah University of Medical Sciences, Kermanshah, Iran; 3 Department of Pharmacy, University of Huddersfield, Queensgate, Huddersfield, UK; 4 Pharmaceutics Research Laboratory, School of Life Sciences, University of Sussex, Brighton, UK

**Keywords:** chitosan, Eudragit RL100, nanofiber, ocular drug delivery, sustained release insert

## Abstract

A novel nanofiber insert was prepared with a modified electrospinning method to enhance the ocular residence time of ofloxacin (OFX) and to provide a sustained release pattern by covering hydrophilic polymers, chitosan/polyvinyl alcohol (CS/PVA) nanofibers, with a hydrophobic polymer, Eudragit RL100 in layers, and by glutaraldehyde (GA) cross-linking of CS-PVA nanofibers for the treatment of infectious conjunctivitis. The morphology of the prepared nanofibers was studied using scanning electron microscopy (SEM). The average fiber diameter was found to be 123 ± 23 nm for the single electrospun nanofiber with no cross-linking (OFX-O). The single nanofibers, cross-linked for 10 h with GA (OFX-OG), had an average fiber diameter of 159 ± 30 nm. The amount of OFX released from the nanofibers was measured *in vitro* and *in vivo* using UV spectroscopy and microbial assay methods against *Staphylococcus aureus*, respectively. The antimicrobial efficiency of OFX formulated in cross-linked and non-cross-linked nanofibers was affirmed by observing the inhibition zones of *Staphylococcus aureus* and *Escherichia coli*. *In vivo* studies using the OFX nanofibrous inserts on a rabbit eye confirmed a sustained release pattern for up to 96 h. It was found that the cross-linking of the nanofibers by GA vapor could reduce the burst release of OFX from OFX-loaded CS/PVA in one layer and multi-layered nanofibers. *In vivo* results showed that the AUC_0–96_ for the nanofibers was 9–20-folds higher compared to the OFX solution. This study thus demonstrates the potential of the nanofiber technology is being utilized to sustained drug release in ocular drug delivery systems.

**Supplementary Information:**

The online version contains supplementary material available at 10.1208/s12249-021-02051-5.

## INTRODUCTION

Bacterial conjunctivitis is a common infection, which occurs in patients of all ages and requires emergency treatment. Ofloxacin (OFX) is a fluoroquinolone antibacterial agent, which is extremely effective against a wide variety of bacteria including Gram-positive and Gram-negative microorganisms by inhibiting DNA gyrase (1, 2). Nanofibers have been studied for ocular drug delivery due to their large surface-to-volume ratio. These systems can also be introduced into the conjunctival sac with effective contact with the ocular tissue. Nanofibers could overcome one of the most important challenges of eye drops which is the limitation of the cul-de-sac volume (∼30 μL) for the administration of eye drop (3, 4).

Chitosan (CS) is a broadly used polymer to design ocular drug delivery systems because of its unique biological properties including antimicrobial activity, biodegradability, and biocompatibility (5). Polyvinyl alcohol (PVA) is a synthetic polymer which is biodegradable, harmless, and has good biocompatible properties (6). Electrospinning of CS and its derivatives is feasible by the addition of a flexible polymer such as PVA which can offer advantageous effects on the biological properties of blend fibers (7, 8). CS/PVA nanofibers dissolve immediately when exposed to water making a substance similar to that of a gelatinous material, which provides an immediate release for the drug used. Therefore, preparing the water-insoluble nanofibrous mat is desirable and is possible by cross-linking the PVA hydroxyl groups with the CS amino groups using chemical agents such as glutaraldehyde, which is a much more suitable cross-linking agent than other aldehydes (9–11).

Several researchers have designed the methods of preparation and evaluation of multi-layered nanofibers including a middle layer with drugs, covered by other layers of various polymeric nanofibers such as Eudragit RL 100 (12–15). There are several publications where researchers have prepared and developed nanofibers for ocular delivery of various drugs. Based on these studies, electrospinning is an efficient method for developing nanofibers compared to other methods like solvent-casting (16, 17). Different kinds of topical ocular formulations of OFX inserts containing HPMC, Eudragit RL-100, and Eudragit L-100 (18), microspheres *in situ* gel (19), nanostructured lipid carriers modified with chitosan oligosaccharide lactate (COL) (20), liposomes (21), microemulsion (22), niosomes (23), chitosan-alginate nanoparticles (24), and poly(-caprolactone) fibers (25) have been reported.

The purpose of this current study was to design and characterize novel mucoadhesive single layer and multilayer nanofibers containing a middle layer prepared by cross-linked or non-cross-linked CS/PVA nanofibers loaded with the potent anti-infective agent, OFX in this case, covered by Eudragit RL100. These novel inserts are expected to prolong drug release, increase ocular availability, and enhance patient compliance by reducing the dosing frequency.

## MATERIALS AND METHODS

### Materials

Chitosan (70–58% deacetylated) was obtained from Acros Organics (Fair Lawn, NJ, USA). Eudragit RL100 and polyvinyl alcohol (PVA) (99% hydrolyzed, average MW = 89–98 kDa) were purchased from Merck (Darmstadt, Germany). Ofloxacin was purchased from Sina Daru (Iran). Acetic acid, glutaraldehyde, and methanol were purchased from Merck (Darmstadt, Germany) and were of analytical grade.

### Preparation of Polymer Solutions and Nanofibers

CS/OFX/PVA solution was prepared by mixing 10 mL of chitosan solution (4% w/v) in acetic acid (1% v/v) with 10 mL of PVA solution (8% w/v) in distilled water and dissolving OFX at 25°C under continuous stirring condition (300 rpm) to obtain a final solution containing 2%, 4%, and 0.6% (w/v) of CS, PVA, and OFX, respectively.

Eudragit RL100 solution (10% w/v) in methanol was obtained using magnetic stirring at 300 rpm at 25°C. The one-layer nanofiber (denoted as the OFX-O formulation) was prepared using an electrospinning device (Fanavaran Nano-Meghyas, Tehran) by loading the CS/OFX/PVA solution in an injector and injecting the solution with a flow rate of 0.5 mL/h under high voltage application (28 kV) toward a cylindrical collector (10 cm diameter). The distance between the injector and the collector was fixed at 15 cm, and the whole process was performed at 25°C and 25% of relative humidity.

**Fig. 1:**
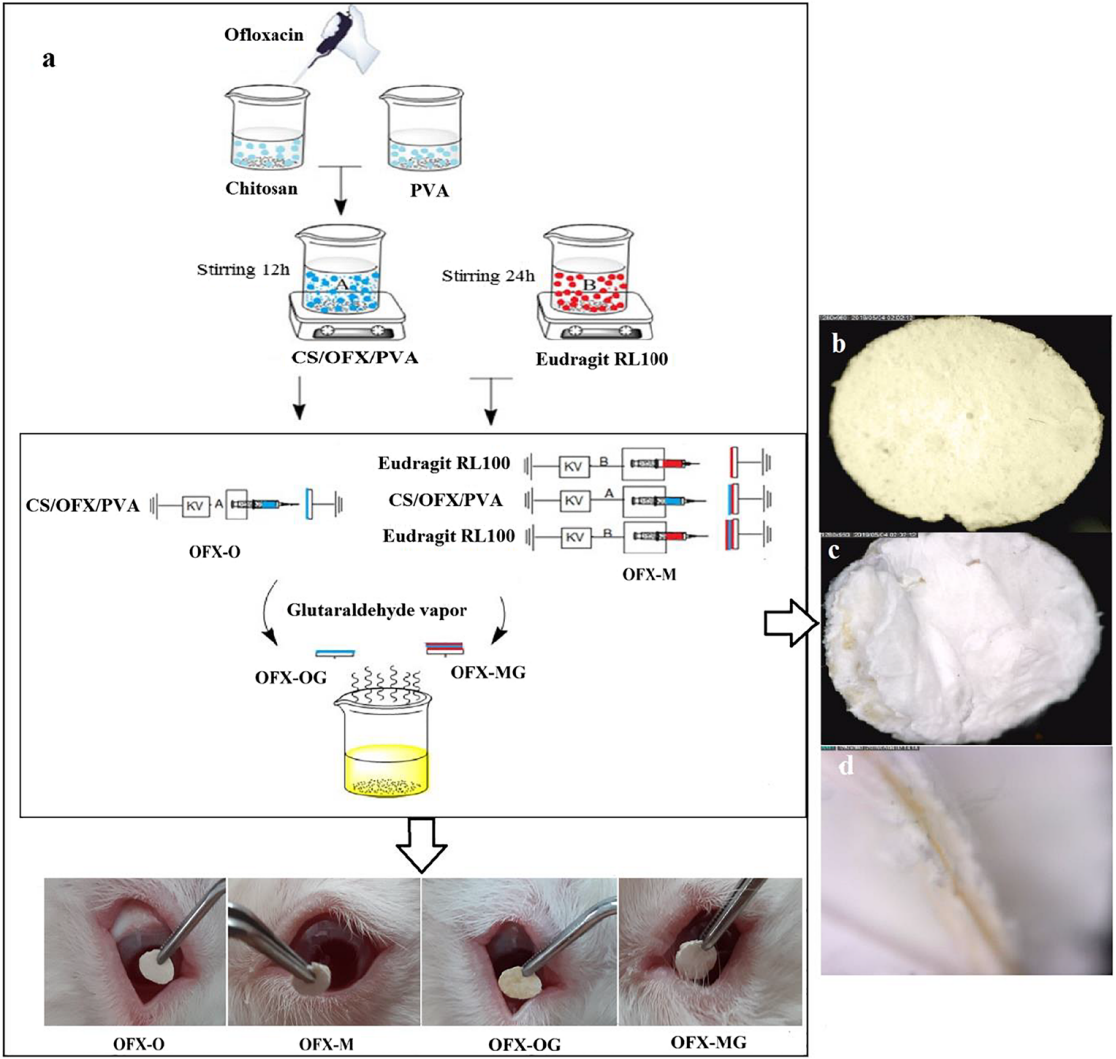
**a** The experimental flow chart of single and multi-layered electrospun nanofibrous structures of different formulations; **b** single-layered electrospun nanofiber (×50 magnification); **c** multi-layered electrospun nanofibrous structures (×50 magnification) and **d** the cross-sectional views of the multi-layered nanofibrous structures (×150 magnification). Note: OFX is ofloxacin; CS is chitosan; PVA is polyvinyl alcohol; OFX-O is single-layered electrospun nanofiber before glutaraldehyde (GA) cross-linking; OFX-M is multi-layered nanofiber before GA cross-linking; OFX-OG is single-layered electrospun nanofiber after GA cross-linking; and OFX-MG is multi-layered nanofiber after GA cross-linking

For the preparation of multi-layered nanofibers (denoted as OFX-M), a specified amount of Eudragit RL100 solution (10% w/v in methanol) was electrospun at a flow rate of 2 mL/h toward a cylindrical collector (10 cm diameter) covered by aluminum foil; then, the core layer was prepared by the spinning of CS/OFX/PVA solution and, lastly, the core layer was covered with another Eudragit RL100 layer. The electrospinning condition maintained the same as the one-layer nanofiber. The experimental flow chart is described in Fig. 1.

The cross-linking procedure was performed using the method as explained by Zhou (26) with some modifications. To cross-link the nanofibers in glutaraldehyde (GA) vapor, the prepared formulations were placed in a desiccator containing 15 mL of aqueous glutaraldehyde solution (50%, v/v) at 25°C for 10 h (these were the OFX-OG and OFX-MG formulations).

### Characterization of Nanofiber Morphology

To investigate the morphology of the nanofibers, the samples were dried at 25°C for 3 h and put on a metal stub using adhesive tape, sputter-coated with gold, and then observed under a scanning electron microscope (FE-SEM, MIRA3, TESCAN, Czech Republic).

### Fourier Transform Infrared Spectroscopy

FTIR spectra were observed by using the FTIR- spectrometer (Shimadzu IR PRESTIGE-21, Japan). A vacuum desiccator was used to dry the nanofibers at room temperature (25°C for 5 h). The samples were thereafter mixed with micronized KBr powder and finally compressed into discs using a manual tablet press.

### Differential Scanning Calorimetry

To investigate the thermal properties of the prepared nanofibers and evaluate the solid-state of the drug in the nanofiber formulations before and after the cross-linking procedure, the DSC analysis was performed. The specimens (around 5 mg) were placed in an aluminum pan. The samples were run under nitrogen gas flow, and the samples were heated from 30 to 500°C with a scanning rate of 5°C/min (DT-Q600 thermal analyzer, TA Instruments Inc., USA).

### Drug Content Uniformity

To evaluate the drug content uniformity, 10-mg samples were dissolved in 5 mL of acetic acid (1% v/v) for single-layered nanofibers and a mixture of acetic acid (1% v/v) and methanol in a 1:1 (v/v) ratio for the multi-layered nanofibers at 100 rpm for 5–6 h to extract OFX from the nanofibers. The drug content was evaluated at a wavelength of 284 nm by the use of a UVmini-1240 spectrophotometer (Shimadzu, Japan) (27). A calibration curve was constructed with an r^2^ value of 0.9994 at a range of OFX concentrations from 0.781 to 25 μg/mL.

### Thickness Measurement

The thickness of the nanofibers (n = 3) was evaluated by a screw gauge with an accuracy of 0.01 mm at various spots of samples.

### Moisture Uptake and Moisture Loss

To evaluate the moisture uptake, the nanofibers were weighed after the electrospinning process and kept in a desiccator with 79.5% relative humidity (RH) (RH was generated by a saturated solution of aluminum chloride in water under ambient temperature). Nanofibers were removed and reweighed after 3 days, and the percentage moisture uptake was calculated (28). To measure the moisture loss percentage, nanofibers were weighed and then kept in a desiccator with anhydrous calcium chloride to avoid any moisture absorption. The samples were reweighed after 3 days, and the moisture loss percentage was estimated (28). The moisture uptake and loss were measured using the following formula:

1 $$ Moisture\ uptake\ and\ loss\ \left(\%\right)=\frac{\left| initial\  wt.- final\  wt.\right|\ }{initial\  wt.}\times 100\kern0.75em $$

### Folding Endurance, Tensile Strength, and Swelling Percentage

Folding endurance was evaluated by frequently folding the 3 × 3-cm^2^ pieces of nanofibers at the same place manually, till it breaks. The folding endurance is the average number (n = 3) of folds in which the nanofibers could be folded at the same place without breaking.

For the determination of tensile strength, one end of the prepared insert (n = 3) was fixed to the movable clip and the other end was fixed to the base plate (Santam STM1, Iran). Using the movable clip which constantly moves upward, a force was gradually added, and the nanofiber was pulled until it was broken (refer to supplementary material Figure S1) (29, 30). The specimen gage length was 20 mm, and the testing rate was fixed at 1 mm/min. The tensile strength was measured from the ultimate load before separation.

To determine the swelling index of the prepared nanofibers, the samples were weighed initially and were then kept in an agar gel plate containing 15 mL of 2% w/v agar at 37 ± 1°C. The nanofibers were reweighed after one hour. The swelling index was calculated based on the following equation:

2 $$ \% Swelling\ Percentage=\frac{wt. of\ swollen\ insert- wt. of\ initial\ insert\ }{wt. of\ initial\ insert}\times 100\kern0.75em $$

### *In Vitro* Antimicrobial Efficacy Test and Sterility Testing

Bactericidal effects of the prepared nanofibers were determined by measuring the inhibitory zone diameter against *Staphylococcus aureus* (*ATCC 6538*) as a Gram-positive organism and *Escherichia coli* (*ATCC 35218*) as a Gram-negative organism. The same swaps were fully soaked with an aliquot (almost 100 μL) of the McFarland standard suspension of microorganism (a McFarland Standard is a chemical solution of barium chloride that is used to standardize the approximate number of bacteria in a liquid suspension by comparing the turbidity of the test suspension), followed by spreading onto an agar plate uniformly (Caso-Agar, Mercoplate®; Merck and Co.). Approximate bacterial suspension per milliliter was 1.5 × 10^8^ CFU/mL. Then, the formulated nanofibers of OFX-O, OFX-M, OFX-OG, and OFX-MG were cut into a diameter of 6 mm were placed on the plates and incubation performed at 35°C for 24 h (31).

The sterility test was carried out under aseptic conditions for the fungi, aerobic bacteria, and anaerobic bacteria utilizing soyabean casein digest medium and thioglycolate broth. Same blank pieces of nanofibers (1 × 1 cm^2^) were immersed in the culture media and incubated at 35°C (25°C for fungi) for 7, 14, and 28 days to investigate any microorganism growth in case of contaminations.

### Microbiological Assay Test

The microbiological assays were performed on *Staphylococcus aureus* (*ATCC 6538*) using the standard disc diffusion method. Tryptic soy agar (TSA) plates were used to cultivate the bacteria. The spread-plate method was utilized (32). The McFarland standard bacterial suspension (almost 100 μL) was spread onto an agar plate uniformly by a soaked swap before placement of the sterile paper discs. Sterile paper discs with a diameter of 5 mm were soaked, 30 μL of the samples along with standard antibiotic containing discs were placed in each plate, and the incubation was performed at 35°C for 24 h. The mean diameter of the inhibition zone surrounding the discs was determined in mm using a Vernier caliper or a scale (0.1 mm) and recorded.

### *In Vitro* Drug Release Study

A simple in-house laboratory *in vitro* drug release study assembly was used to simulate the conditions of the ocular cavity. The various nanofiber formulations (OFX-O, OFX-M, OFX-OG, or OFX-MG) were placed inside a donor compartment containing 25 mL of pH 7.4 phosphate-buffered saline (PBS). A dialysis membrane (cut off diameter 12,000 Da) was tied at one end of the donor compartment. Then, it was placed from the tied end in the receptor compartment contained 25 mL of the same buffer (to preserve the sink condition). A volume of 2 mL of the OFX content diffused from the dialysis membrane from the tested formulation sample was taken at different time intervals from the receptor compartment for analysis. This was replaced immediately with a volume of 2 mL fresh PBS to maintain the sink condition. The released drug (OFX) was measured using a UV-Vis spectrophotometer (Shimadzu, Japan) at a wavelength of 284 nm.

### *In Vitro* Cytotoxicity Test

Cytotoxicity of the nanofibrous inserts was determined against L929 (mouse fibroblast). Firstly, the cells were added to each well of a 96-well tissue culture plate at a density of 4 × 10^5^ cells/mL in 200 μL medium per well for 72 h. Dulbecco’s modified Eagle’s medium (DMEM/F12), medium (1:1 v/v, Gibco, Paisley, Strathclyde, UK), supplemented with 10% fetal bovine serum (Gibco Invitrogen S.r.l., Milan, Italy), 100 U/mL penicillin, and 100 μg/mL streptomycin in a humidified incubator at 37°C with 5% CO_2_ were used.

A certain row of 24-well plates without installation of nanofibers was considered as the control. OFX nanofibers with various concentrations of OFX in the medium (corresponding to 12.5, 25, 50, or 100 μg/mL OFX) were placed in the other wells. Thirty microliters of the medium and 3-(4,5-dimethylthiazol-2-yl)-2,5-diphenyl-2H-tetrazolium bromide (MTT) assay was placed into all of the wells and incubated for 4 h. Of DMSO solution, 150 μL was placed into the wells and the plate observed by the microplate reader. The absorbance ratio of sample cells to control cells measured at the wavelength of 560 nm was then calculated. All experiments were carried out in triplicate (mean ± SD, n = 3).

### *In Vivo* Studies and Ocular Irritation Study on Rabbit Eyes

To evaluate the irritancy of nanofibers, the Draize test was adopted. One of the rabbits’ eyes received sterile inserts (inserts were prepared in aseptic conditions followed by 40 min exposure to UV light) formulations (OFX-O, OFX-M, OFX-OG, or OFX-MG), while the other eye was treated with sterile PBS as a control for. The eyes were observed for 7 days for any sign of irritancy including abnormal discharge, congestion, and redness of the conjunctiva. Corneal opacity and irritation were graded using the scoring system considered in guidelines for ocular irritation study between 0 and 2 showing the intensity of irritation (33).

Permission for the use of animals was obtained from the animals’ ethics committee of Kermanshah University of Medical Sciences (Approval No. IR.KUMS.REC.1396.305). Twelve New Zealand white rabbits weighing 3.8–4.1 kg were used in the experimental and control groups. A punch of nanofibrous inserts (estimated to contain about 20 mg for single-layered and 60 mg for multi-layered nanofibers, respectively) and OFX solution with a drug content of 3% w/v for multi-layer and 9% w/v for one-layer nanofibers used as the standard was introduced into the rabbit’s conjunctival sac. At the time of sampling, 50 μL of sterile PBS was poured into the eyes of the rabbit and the tears collected by the sterile paper discs. These discs were transferred directly onto the culture medium, and the amount of drug remaining in the paper disks was measured by the microbial assay method as explained earlier.

## RESULTS AND DISCUSSION

### Evaluation of Ocular Inserts

CS/PVA nanofibers were successfully manufactured by electrospinning. The process of manufacturing the ocular inserts with single-layered and multi-layered nanofibrous structures is demonstrated in Fig. 1. The amount of the loaded OFX in the nanofibers was 3–9% w/w which is higher than that of commercial conventional (eye drop formulation with 0.3% w/w).

It was interesting to note that the entrapment efficiency was found to be > 95% for all the 4 formulations (OFX-O, OFX-M, OFX-OG, or OFX-MG) (Table I). The high entrapment efficiency is attributed to the technique used which involves the incorporation of OFX into the CS/PVA nanofibers. Electrospinning leads to the formation of fibers with higher free space and porosities between nanofibers which results in higher entrapment efficiency which is required for the sustain release pattern (34). This high drug entrapment efficiency allows using smaller inserts which can enhance patient compliance for self-administration of the inserts.

**Table I:** Physicochemical Parameters of the Ocular Inserts of Ofloxacin Nanofibers (Mean ± SD, n = 3)

Formulation	Thickness (mm)	Folding endurance	Tensile strength (MPa)	Entrapment efficiency (%)	Swelling (%)	Moisture loss (%)	Moisture uptake (%)
OFX-O	0.084 ± 0.004	183 ± 3	2.4 ± 0.2	95.3 ± 0.8	136.3 ± 4.5	1.24 ± 0.05	1.12 ± 0.02
OFX-M	0.095 ± 0.002	209 ± 2	10.6 ± 1.0	97.7 ± 0.9	116.9 ± 2.3	1.16 ± 0.02	9.05 ± 0.04
OFX-OG	0.075 ± 0.002	194 ± 5	3.2 ± 0.5	96.3 ± 0.7	107.5 ± 2.8	0.90 ± 0.02	0.64 ± 0.06
OFX-MG	0.093 ± 0.002	214 ± 5	11.7 ± 1.2	98.9 ± 0.6	92.5 ± 3.9	0.67 ± 0.02	0.52 ± 0.01

*OFX-O* single-layered electrospun nanofiber before GA cross-linking, *OFX-OG* single electrospun nanofiber after GA cross-linking, *OFX-M* multi-layered nanofiber before glutaraldehyde (GA) cross-linking, *OFX-MG* is multi-layered nanofiber after glutaraldehyde (GA) cross-linking

The physicochemical parameters related to the prepared OFX inserts like thickness, swelling, moisture uptake, moisture loss, folding endurance, and tensile strength were also evaluated and displayed in Table I. The nanofibers had a suitable thickness from 0.075 ± 0.002 to 0.095 ± 0.002 mm. As reported in previous studies, commercially manufactured ocular inserts of Ocusert® possessed 0.300 mm thickness (33). Hence, the prepared nanofibers with less than 0.1 mm thickness were expected to be non-irritant to the eyes over ocular administration. All the prepared nanofibers were of a suitable thickness and hence expected not to irritate the eyes over ocular administration due to a higher thickness. The OFX nanofibers showed appropriate folding endurance, which indicated that these systems are adequately flexible and can simply be inserted into the conjunctival sac. The OFX nanofiber inserts with GA vapor exposure showed higher flexibility and suggests a potential link between the cross-linking of CS/PVA nanofibers and increasing their strength. The enhanced ultimate flexibility showed that cross-linking by GA vapor can make CS/PVA nanofibers more stable and mechanically strong. In fact, having a rigid web because of strong inter-fiber bonding between nanofibrous structures could often happen at the intersection points. This can enhance the physical and mechanical characteristics of the cross-linked fibers (35, 36). The reported tensile strength for ocular formulations was between 1 and 30 MPa for different formulations. The OFX-MG nanofiber showed the highest tensile strength. The reason behind the increased flexibility could be due to the presence of the CS/PVA layer made from cross-linked nanofibers and the presence of covering layers which were made from the Eudragit nanofibers (14).

The degree of the swelling has a significant effect on the drug-releasing behavior of the nanofibers. The swelling degree was lower in the cross-linked nanofibers (OFXOG and OFX-MG) compared to the non-cross-linked counterparts (OFX-O and OFX-M). This pattern could be due to the cross-linking of chitosan, PVA, and CS/PVA and the enhancement of intermolecular bonding which result in the reduction of the degree of swelling, and the rate of drug release (36) Furthermore, Eudragit RL100 nanofibrous layers are relatively hydrophobic in nature, hence the lower amount of tear fluid absorbed leading to the decreased degree of swelling observed in the multi-layered nanofibers compared to single-layered nanofibers. Moisture uptake percentages were in the range of 0.52 ± 0.01 to 1.12 ± 0.02%, and the amount of moisture loss was found in the range of 0.67 ± 0.02 to 1.24 ± 0.05% (Table I). Based on these results, it was revealed that the prepared nanofibers have good physical stability in conditions with various moisture contents.

### Fourier Transform Infrared Spectroscopy

**Fig. 2:**
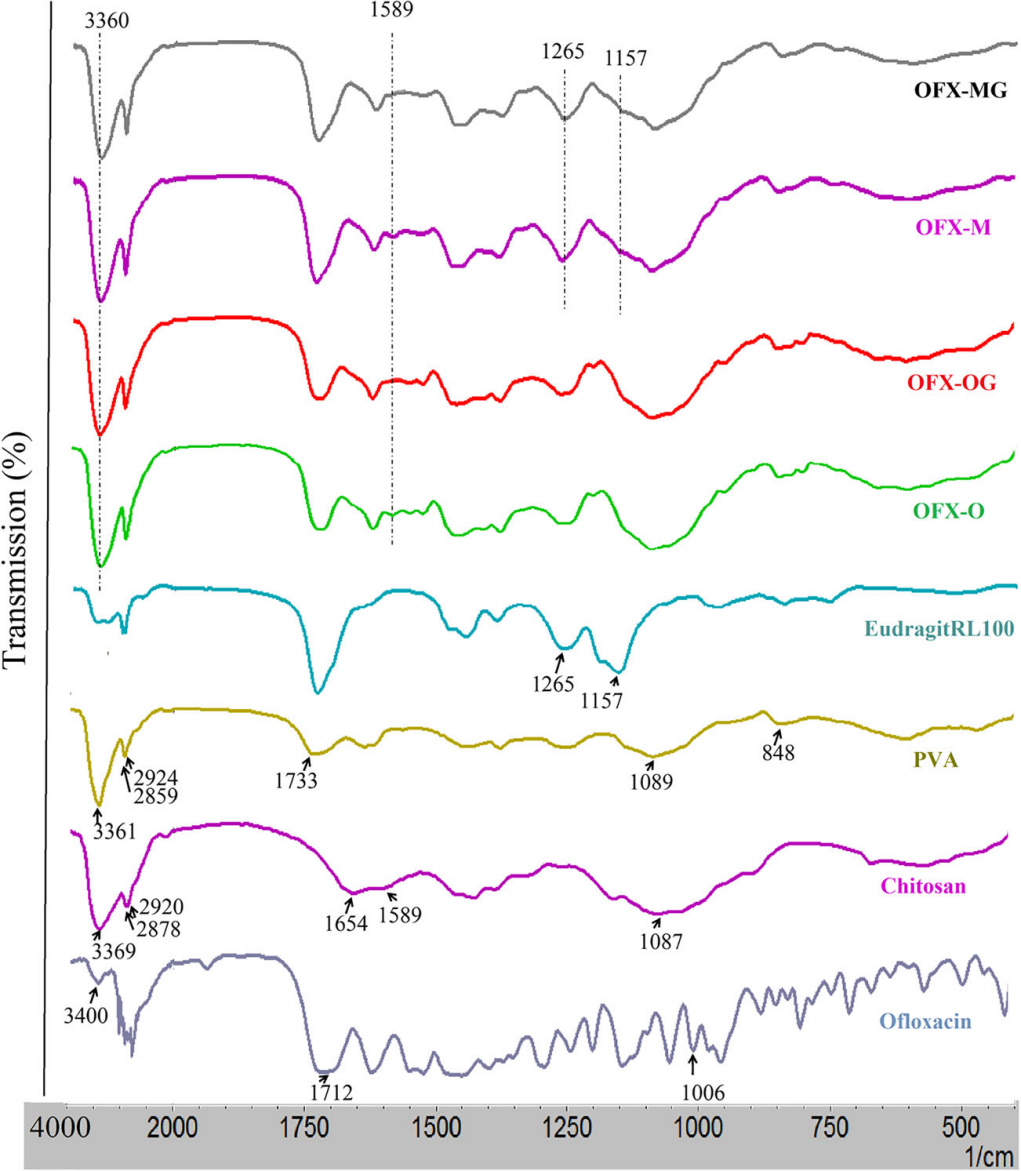
FTIR spectra of chitosan, PVA, ofloxacin, Eudragit RL100, OFX-O, OFX-OG, OFX-M, and OFX-MG nanofibers. Note: PVA is polyvinyl alcohol; OFX-O is single-layered electrospun nanofiber before GA cross-linking; OFX-OG is single-layered electrospun nanofiber after GA cross-linking; OFX-M is multi-layered nanofiber before glutaraldehyde (GA) cross-linking; and OFX-MG is multi-layered nanofiber after glutaraldehyde (GA) cross-linking

The FTIR spectra of the OFX nanofibers, chitosan, PVA, and ofloxacin are shown in Fig. 2. The FTIR spectrum of ofloxacin showed its characteristic peaks at ~1006 cm^−1^ for C-F, 1712 cm^−1^ for C=O, and 3400 cm^−1^ for OH. The appearance of OFX characteristic peaks in the nanofibers confirmed the presence of ofloxacin inside in nanofibers structure. Some characteristic bands for PVA and chitosan in the prepared OFX nanofibers were consistent with the literature data (37). The vibrational peak appearing at 848.68 cm^−1^ was attributed to C–H rocking mode of PVA and also the absorption peak at 1654 cm^−1^ was assigned to the amide type I of chitosan. ACH and CH symmetric and asymmetric stretching vibrations for OFX nanofibers appeared at 2859 cm^−1^ and 2927 cm^−1^. The absorption bands in the FTIR spectra of chitosan/PVA nanofibers for OH and NH appeared at 3360 cm^−1^ and 1589 cm^−1^, which confirmed the structure of the CS/PVA nanofibers with hydrogen bonding (38, 39).

Decreasing of the peak intensity at 3360 cm^−1^ of cross-linked nanofibers (OFX-OG and OFX-MG) occurred with the cross-linking reaction which could be due to the reduction of amino and hydroxyl groups. The peaks at 1598 cm^−1^ related to NH_2_ were mostly eliminated for the cross-linked OFX nanofibers which are indications of engaging the amino group in chitosan following GA cross-linking. These results indicated the successful occurrence of cross-linking reaction between PVA/CS nanofibrous structure and GA (40, 41).

The spectra produced by FTIR for the Eudragit RL100 are also presented in Fig. 2. It can be observed that there are strong bands at 1157 cm^–1^ and 1265 cm^–1^ due to the stretch of carbonyl (ester) groups present in the Eudragit. In the multi-layered nanofibers (OFX-M and OFX-MG), the ester vibration group of Eudragit RL100 was observed at 1157 and 1261 cm^−1^.

### Differential Scanning Calorimetry

**Fig. 3:**
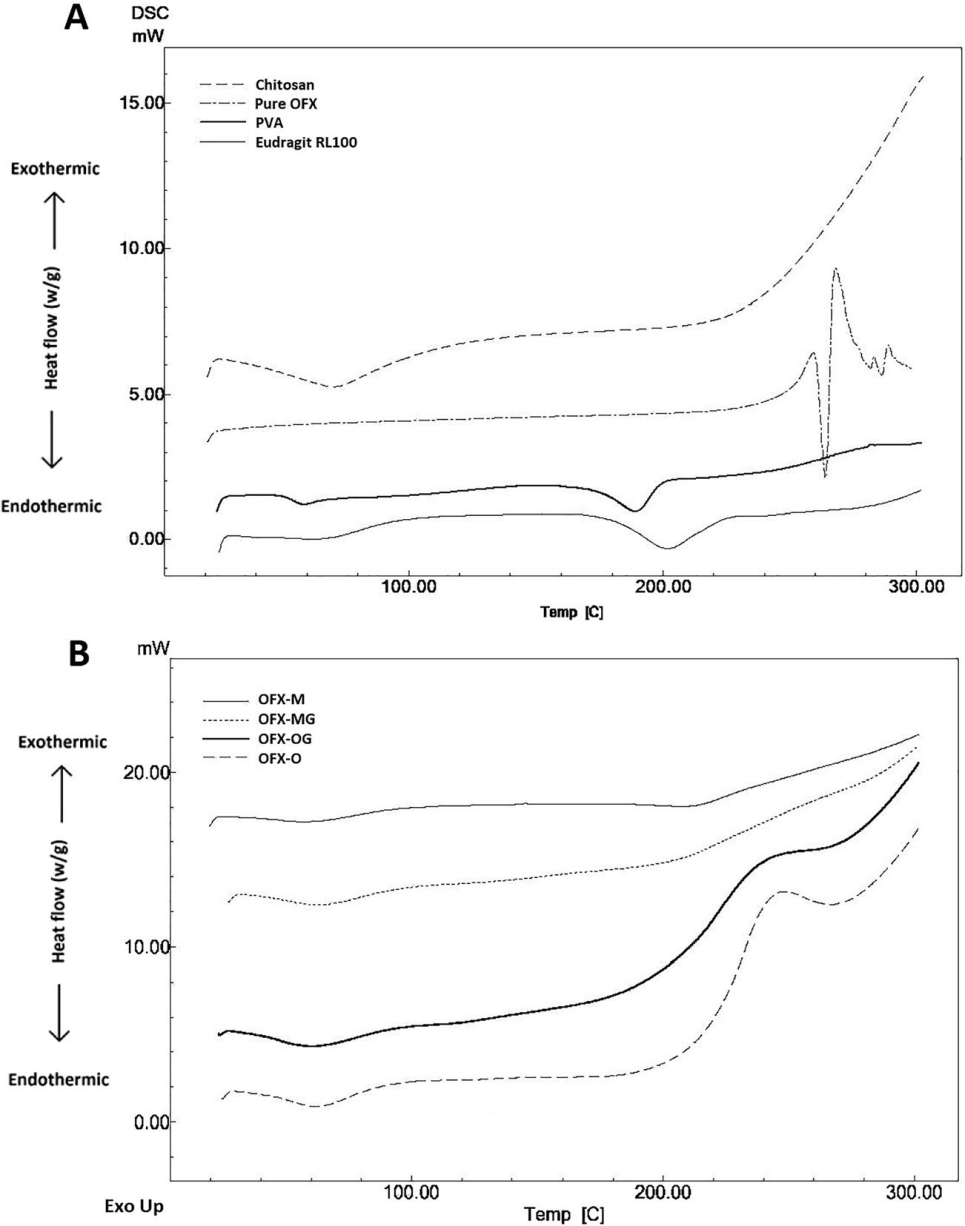
DSC traces of **a** all excipients used in the preparation of nanofibers, **b** non-cross-linked (OFX-O and OFX-M), and cross-linked (OFX-OG and OFXMG)formulations

The DSC analysis was carried out to observe the phase transition and thermal properties of drug and polymers in the nanofibrous structures. The DSC traces of all excipients and materials used in the preparation of nanofibers are shown in Fig. 3A. The thermograph of PVA showed the glass transition at 59°C and the melting peak of PVA at 190°C (42). Pure chitosan exhibited a broad endothermic peak at around 70°C related to loss of water and an exothermic peak at around 300°C related to chemical decomposition (43). Eudragit DSC traces showed glass transition temperature at around 65°C (44). The DSC traces of pure drug (OFX) showed an endothermic peak around 273°C which is its melting point followed by several small endothermic peaks which could be due to the degradation of the drug at high temperature. Figure 3 B shows that the sharp endothermic peak of OFX in nanofibers is not detectable which could be due to the molecular dispersion of the drug in the nanofibers or the amorphous state of the drug in nanofibers (25). In addition, the endothermic peak observed for pure PVA at 200 C is disappeared from the DESC traces of nanofibers which could be due to the non-crystalline structure of nanofibers formed as a result of fast solidification in the electrospinning (45, 46).

### Characterization of Nanofiber Morphology

**Fig. 4:**
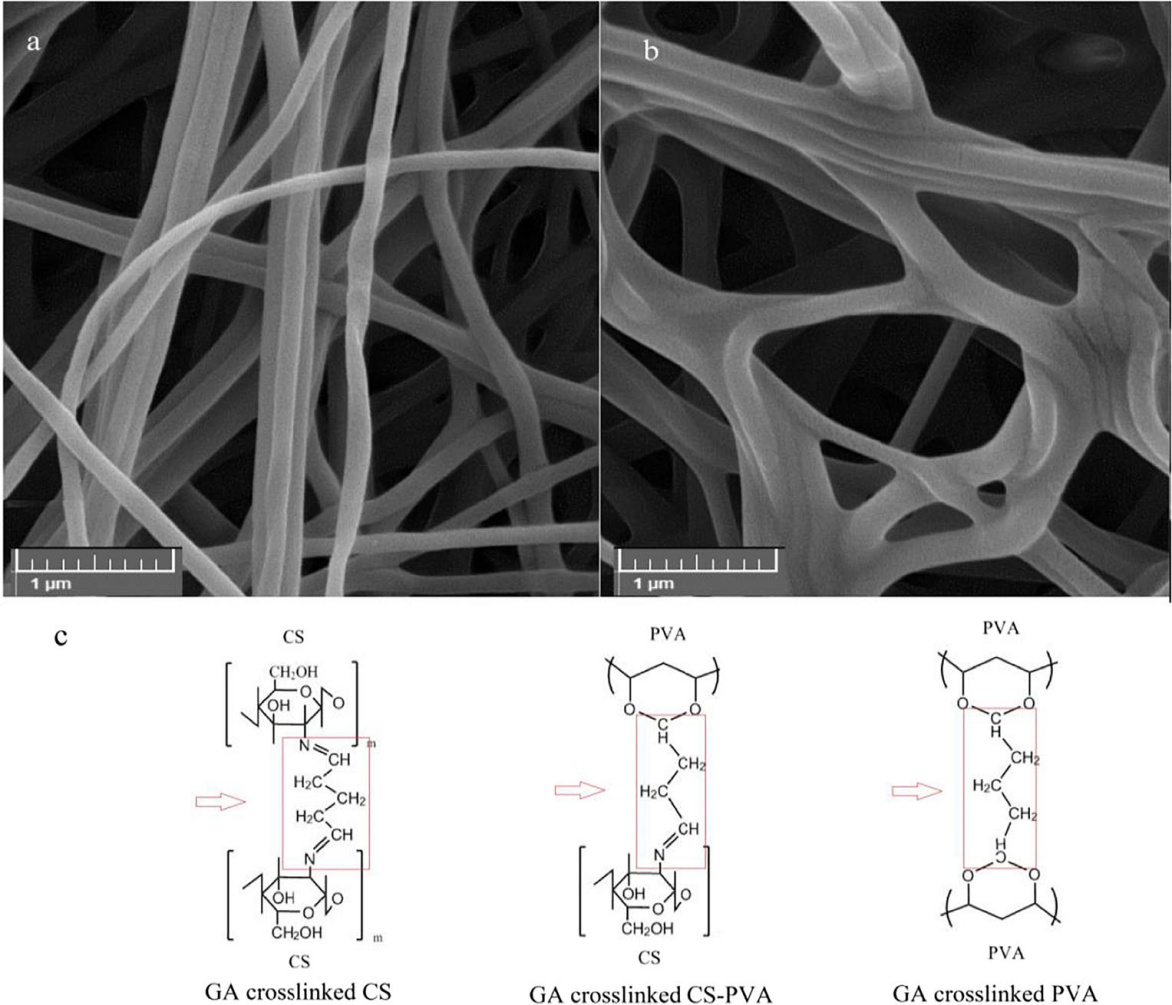
SEM images of CS/PVA nanofibers **a** before GA cross-linking (OFX-O formulation) and **b** after GA cross-linking (OFX-OG formulation) and **c** schematic representation of cross-linking reactions form bonds between chitosan, PVA, and GA

SEM images of CS/PVA nanofibers before (OFX-O) and after GA cross-linking (OFX-OG) are shown in Fig. 4. As seen in Fig. 4, continuous, uniform nanofibrous structures (no beads), and randomly oriented fibers were obtained. The cross-linked nanofibers (159 ± 30 nm) had a greater diameter compared to the non-cross-linked nanofibers (123 ± 23 nm). The reason behind the increased diameter seems to be the swelling of the nanofibers during the cross-linking process with the GA (Fig. 4 a and b). The cross-linking reaction forms covalent bonds between chitosan-chitosan, chitosan-PVA, and PVA-PVA by the GA (Fig. 4c).

**Fig. 5:**
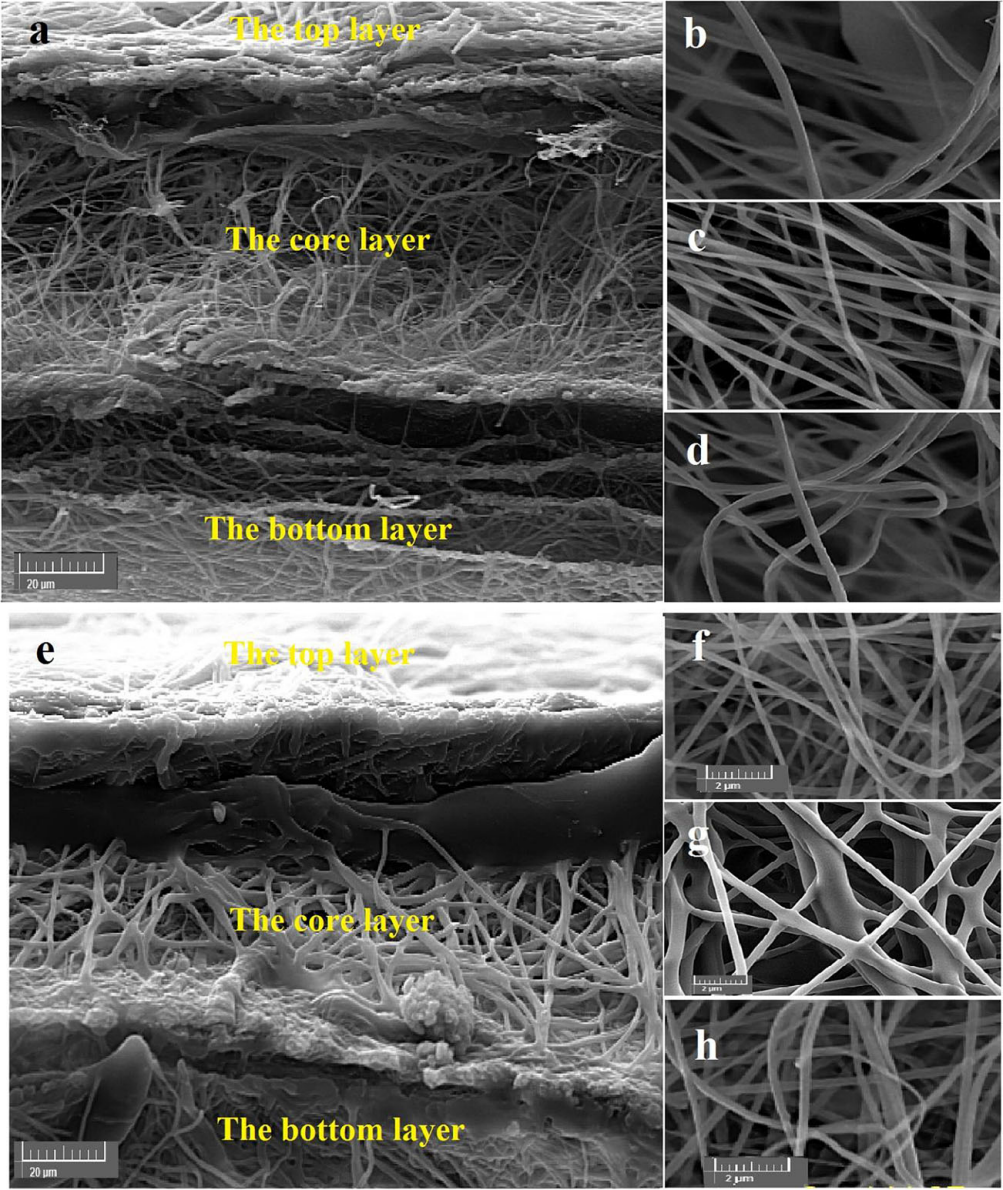
SEM images of multi-layered electrospun nanofibrous structures before GA cross-linking (OFX-M formulation): **a** the cross-section views of multi-layered electrospun nanofibrous structures, **b **top Eudragit RL100 layer, **c** CS-PVA-OFX at the core, **d** bottom Eudragit RL100 layer, **e** the cross-section of multi-layered electrospun nanofibrous structures after GA cross-linking (OFX-MG formulation), **f** top Eudragit RL100 layer, **g** CS-PVA-OFX at the core, and **h** bottom Eudragit RL100 layer

The multi-layered nanofibers before the cross-link (OFX-M) and after cross-link (OFX-MG) are shown in Fig. 5. All SEM images showed bead free structure. Comparing all the layers before cross-linking for OFX-M showed that the top layer which contains Eudragit RL100 (Fig. 5(b)) has a higher average diameter in comparison to the core CS/PVA layer (Fig. 5(c)) and bottom layer (Fig. 5(d)). In the case of OFX-MOG (after cross-linking with GA), the core layer (Fig. 5(g)) showed a higher average diameter compared to top (Fig. 5(f)) and bottom layers (Fig. 5(h)).

### *In Vitro* Cytotoxicity Test

**Fig. 6:**
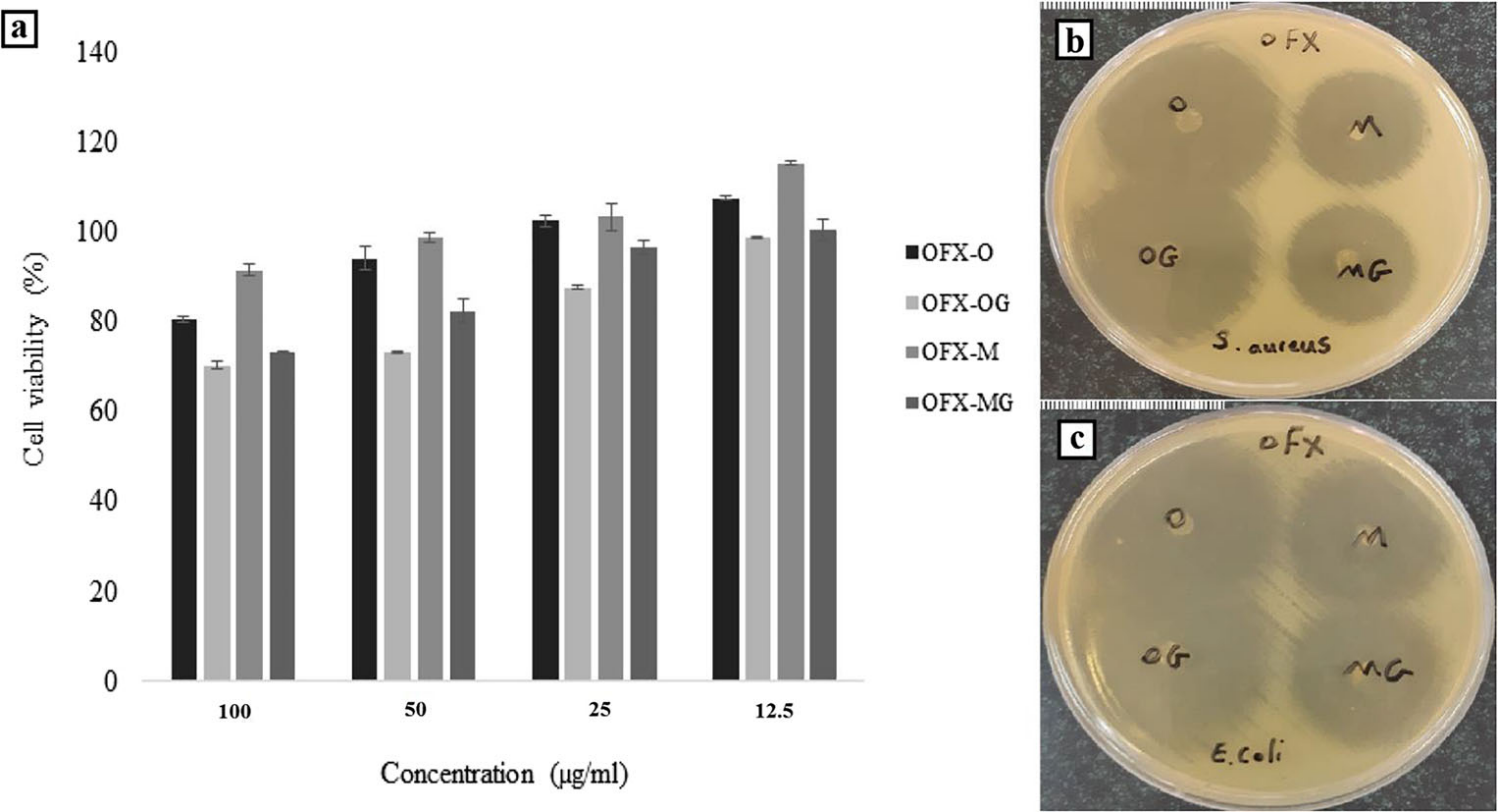
**a** Cell viability after treatment with OFX nanofibers (single-layered (OFX-O, OFX-OG) and multilayered (OFX-M, OFX-MG) electrospun nanofibers) with various concentrations of OFX in medium (corresponding to 12.5, 25, 50, or 100 μg/mL OFX) (n = 6, mean ± SD). Inhibition growth area of singlelayered (OFX-O, OFX-OG) and multi-layered (OFX-M, OFX-MG) electrospun nanofibers; areas of inhibited growth of **b** *Staphylococcus* *aureus* and **c ***Escherichia coli*; note: scale 1 mm

The results indicated that the viability of L929 cells (mouse fibroblasts) could decrease with increasing OFX concentration in the OFX nanofibers formulations (OFX-O, OFX-M, OFX-OG, or OFX-MG) (Fig. 6a). As a result, the nanofibers after GA cross-linking indicated relatively reduced cell viability in comparison with the non-cross-linked nanofibers. Although the nanofibers were washed, it seems that due to the presence of the trace amount of residual GA on the surface of cross-linked nanofibers, the viability was decreased.

Based on the previous studies, the cell viability > 70% could be an indication of non-irritant formulations (47). OFX-OM showed less reduction in cell viability in comparison with OFX-O because of covering CS/PVA layer by Eudragit RL100 layers. This study showed that the viability of cells was more than 70% in all of OFX nanofibers, with various concentrations of OFX in the medium (corresponding to 12.5, 25, 50, or 100 μg/mL OFX); hence, OFX nanofibers can be utilized for ocular drug delivery as a safe carrier system due to low cell cytotoxicity and good biocompatibility of the prepared nanofibers.

### *In Vitro* Antimicrobial Efficacy Test and Sterility Testing

The inhibitory zones for the nanofibers are shown in Fig. 6 b and c. The average inhibitory zone for OFX-M, OFX-O, OFX-MG, and OFX-OG was measured to be 28 ± 1, 39 ± 1, 29 ± 1, and 40 ± 2 mm for *S. aureus* and 36 ± 1, 41 ± 1, 35 ± 1, and 41 ± 1 for *E. coli*, respectively.

The results showed clear inhibitory zones to be found around all of the nanofibers for both *S. aureus* and *E. coli*. There were bigger inhibitory zones on *E. coli* cultures compared to *S. aureus*, however, the inhibitory efficacy of OFX against *S. aureus* was still retained (Fig. 6b, c). The antibacterial effect of GA on *E. coli* activity has been reported (48). This test also showed that there were no significant differences between the antibacterial activity of cross-linked nanofibers and non-cross-linked nanofibers. This may be because most of the groups with antibacterial activity in CS did not react with the aldehyde groups of GA, thus preserving their antibacterial efficacy.

A previous study reported that cross-linked CS/PVA with GA had suitable antibacterial efficacy for applications in drug delivery (49). Smaller inhibitory zones were observed on *S. aureus* cultures and *E. coli* for multi-layered nanofibers (OFX-M, OFX-MG formulations) compared to single-layered nanofibers (OFX-O, OFX-OG). These smaller inhibitory zones may be due to the covering of the CS/PVA layer containing antibiotics by Eudragit RL100 layers and the reduction of OFX release rate as a result. The OFX nanofibers were sterilized by UV radiation, and sterility testing was performed under aseptic conditions; as such, there was no evidence of the growth of fungi and bacteria in the culture media.

### *In Vitro* Drug Release Study

**Fig. 7:**
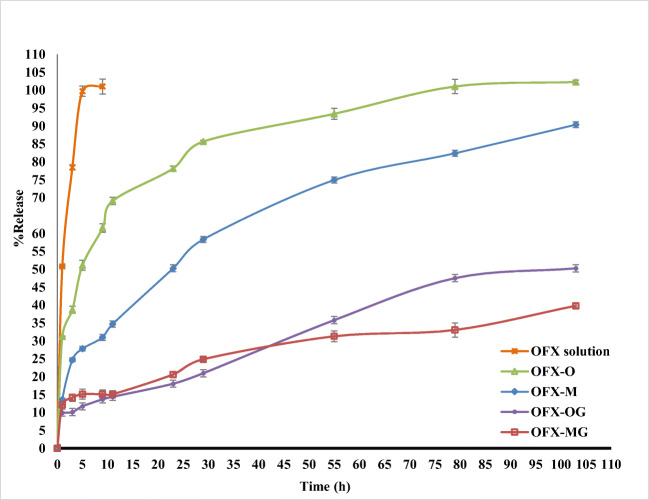
*In vitro* cumulative release behavior of ofloxacin from the various formulations: OFX-O (single-layered electrospun nanofiber before glutaraldehyde (GA) cross-linking), OFX-M (multi-layered nanofiber before GA cross-linking), OFX-OG (single electrospun nanofiber after GA cross-linking), and OFX-MG (multi-layered nanofiber after GA cross-linking)

The results indicated that OFX nanofibers can control drug release up to about 103 h (Fig. 7). The OFX-O formulation containing the non-cross-linked CS/PVA one-layered showed nearly 93.8% drug release, and OFX-M formulation containing the non-cross-linked CS/PVA multi-layered showed nearly 84.17% release of the drug. This value was much smaller for OFX-OG containing the cross-linked CS/PVA one-layered (50.26%) and OFX-MG containing the cross-linked CS-PVA multi-layered nanofibers (39.82%) at the end of 103 hours (Fig. 7).

Two important factors affected the prolongation of the OFX release from inserts: (1) covering the CS/PVA layer containing the OFX drug by the Eudragit RL100 layers and (2) GA cross-linking the CS/PVA nanofibers. In the case of CS/PVA nanofibers, the immersion in tear fluid causes the instant dissolution of the nanofiber into a substance similar to that of gelatinous material. This can therefore limit the usage of a CS/PVA layer in the eye tissues. To improve its mechanical properties, chemical cross-linking with GA vapor was utilized due to the bonds formed between the reactive side groups of CS (amino groups) and PVA (hydroxyl groups) (50, 51). This reaction occurred from the surface to the inside of the layers by the GA. The resultant intermolecular forces, therefore, leads to a reduction in the swelling pace and also the drug delivery rate (51).

Eudragit RL100 polymer can thus effectively provide prolonged and sustained release of OFX from multi-layered nanofibers (OFX-M, OFX-MG formulations). Indeed, combinations of hydrophilic and hydrophobic nanofiber layers can control the drug release and therefore reduce the toxicity as a result of the fast release of drugs (13). These nanofibrous structures can be useful for the management of ocular bacterial infections when the prolonged release of an antibiotic is essential. Although the identical formulations had not been prepared in any previous studies, a similar study has designed and prepared OFX-loaded chitosan/PVA nanofibers for hernia repair (42). They showed a similar two-phase release of OFX from nanofiber during 28 days with a burst release in the first 8 h (52). For the ophthalmic anti-infective formulations, 1 week of drug release could be suitable.

### Ocular Irritation and *In Vivo* Studies

During the Draize irritancy test, no symptoms of ocular irritation such as inflammation, the opacity of the cornea, conjunctival redness, and discharge were observed. Redness of conjunctivae was however observed after administration of the cross-linked nanofibers with GA, which was reduced and vanished over time. There is either negligible or no irritation and inflammation of the eye for all the tested eyes with all the formulations compared to control eyes which suggested that the OFX nanofibers were well-tolerated.

The OFX nanofibers were inserted into the rabbits' conjunctival sac without the application of any invasive method. The concentration of released OFX from nanofibers in the tear fluid was determined by microbiological assay (*Staphylococcus aureus*) using the standard curve. This method was reproducible, linear, precise, and simple. Moreover, this method does not require toxic solvents and specialized equipment. Drug released from nanofibers (96 h reading) and pure commercial OFX standard demonstrated substantially similar clear inhibitory zones.

**Fig. 8:**
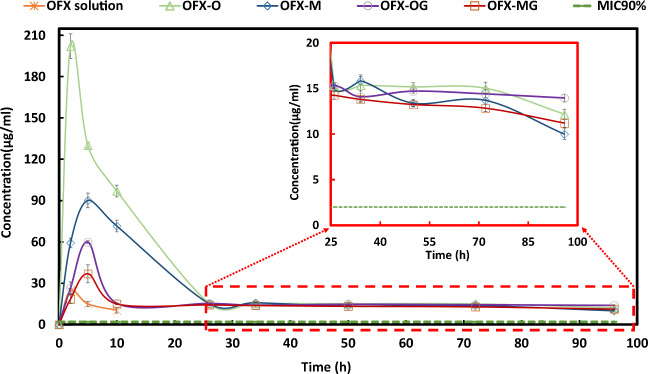
Plots of *in vivo* concentration (μg/mL) drug release for the various formulations: OFX-O (single-layered electrospun nanofibers before GA cross-linking), OFX-M (multi-layered nanofibers before GA cross-linking), OFX-OG (single-layered electrospun nanofibers after GA cross-linking), OFX-MG (multi-layered nanofibers after GA cross-linking). Dotted green line is the minimum inhibitory concentration of OFX against microorganisms (MIC90%). Red insert shows an expanded view of the subtle differences in the formulations post 25 min upon administration

The lower limit of quantitation for OFX was 7.8 μg/mL. The measured concentration of OFX solution after 2 h was 24 ± 2 μg/mL. However, the measured concentrations were reported to reach below the limit of quantification (LOQ) after 10 h. Maximum concentrations (C_max_) of 60 ± 4 μg/mL and 37 ± 6 μg/mL were measured at 5 h after OFX-OG and OFX-MG administration, respectively, followed by a steady release in tear fluid for 96 h. The non-cross-linked OFX nanofibers indicated a maximum concentration (C_max_ = 202 ± 15 μg/mL (OFX-O) and 90 ± 5 μg/mL (OFX-M)) followed by a steady release in tear fluid for 96 h (Fig. 8). These were significantly higher compared to the cross-linked OFX nanofibers. OFX-O demonstrated the highest C_max_ and shortest T_max_ compared to the other formulations (Table II), which led to the highest rate of absorption. The lowest C_max_ was related to OFX solution, which could be attributed to the fast removal of drugs from the eye surface and decreased corneal contact time for the drug from OFX solution.

**Table II:** Pharmacokinetic Parameters in Rabbit Tears After Drop Instillation of 50 μL of AZM Solution or OFX Fibers with Different Formulations (mean ± SD, n = 6)

Formulation	C_max_ (μg/mL)	T_max_ (h)	AUC_0–96_ (μg h/mL)	MRT (h)
OFX-O	202 ± 15	2	3191 ± 117	24.5 ± 0.1
OFX-M	90 ± 5	5	2320 ± 60	28.8 ± 0.4
OFX-OG	60 ± 4	5	1597 ± 32	41.7 ± 0.5
OFX-MG	37 ± 6	5	1361 ± 20	43.4 ± 0.8
OFX Solution	24 ± 2	2	147 ± 4	4.7 ± 0.1

*OFX-O* single-layered electrospun nanofiber before GA cross-linking, *OFX-OG* single electrospun nanofiber after GA cross-linking, *OFX-M* multi-layered nanofiber before glutaraldehyde (GA) cross-linking, *OFX-MG* is multi-layered nanofiber after glutaraldehyde (GA) cross-linking, *MRT* mean residence time

AUC_0–96_ of the drug in OFX-O, OFX-M, OFX-OG, OFX-MG, and OFX solution was 3191 ± 117, 2320 ± 60, 1597 ± 32, 1361 ± 20, and 147 ± 4 μg h/mL, respectively. These results indicated that the cross-linked OFX nanofibers containing OFX-OG and OFX-MG showed a 10.83-fold and 9.23-fold increase in the AUC_0–96_ compared with OFX solution, respectively. While the non-cross-linked OFX nanofibers containing OFX-O and OFX-M showed a 21.64-fold and 15.74-fold increase in AUC_0–96_ compared to the OFX solution, respectively. The mean residence time (MRT) of OFX-MG was enhanced significantly compared to the other formulations and the OFX solution. This was attributed to the covering of the CS/PVA layer containing the drug by the Eudragit RL100 layers and GA cross-linking.

The minimum inhibitory concentration (MIC90%) of OFX against microorganisms which is infective to the eye including Gram-negative and Gram-positive was reported to be 2 μg/mL (53). While the OFX solution only achieved tear concentrations higher than the MIC90% for the 10 h after instillation, OFX nanofibers remained higher than this level for 95 h. The level of OFX was 9.98–13.96 μg/mL for OFX nanofibers after 95 h after administration, which was 4.99- to 6.98-fold over the MIC. In fact, the prolonged drug release in the precorneal tissue was observed through utilizing the developed nanofibers. These carriers could reduce the frequency of administration of OFX along with lowering the required dose for achieving suitable therapeutic concentrations compared to OFX solution. Similar to the current study, a polycaprolactone-based nanofibrous formulation of levofloxacin designed for ophthalmic administration has released the drug in 30 days in the rat’s eye. The longer release period is due to the more hydrophobic nature of the polycaprolactone (54).

## CONCLUSION

In the present study, electrospinning technology was successfully used for the preparation of single-layered and multi-layered nanofibers with OFX incorporated into the CS/PVA layers. Morphological characterization of the ofloxacin nanofibers revealed highly homogeneous nanofibrous structures with a tight connection between the individual nanofiber layers. Glutaraldehyde cross-linked and non-cross-linked nanofibers for all formulations had an acceptable level of toxicity on cultured L929 (mouse fibroblast) cells. The OFX nanofibers could be placed in the cul-de-sac of the rabbit eyes as an insert without any invasive methods or surgery. There was not any sign of significant inflammation or redness in the rabbits’ eyes. The drug release profile was evaluated by a microbiological assay method using sterile paper discs which are a non-invasive, cost-effective, and a simple method. The developed OFX nanofibers formulations effectively retained the drug concentration in the tear fluid of rabbits above the MIC90% for up to 95 h. This eliminates the need for frequent installation of the drug and enhances patient compliance.

## Supplementary Information

ESM 1 (DOCX 774 kb)
