# Supplementary material for: Polyvinyl Alcohol/Chitosan Single-Layered and Polyvinyl Alcohol/Chitosan/Eudragit RL100 Multi-layered Electrospun Nanofibers as an Ocular Matrix for the Controlled Release of Ofloxacin: an In Vitro and In Vivo Evaluation
Source: AAPS PharmSciTech. 2021 Jun 3;22(5):170. doi: 10.1208/s12249-021-02051-5 (PMC8175245; doi:10.1208/s12249-021-02051-5)
Supplement: Supplementary file 1 — (DOCX 774 kb) [file 12249_2021_2051_MOESM1_ESM.docx]

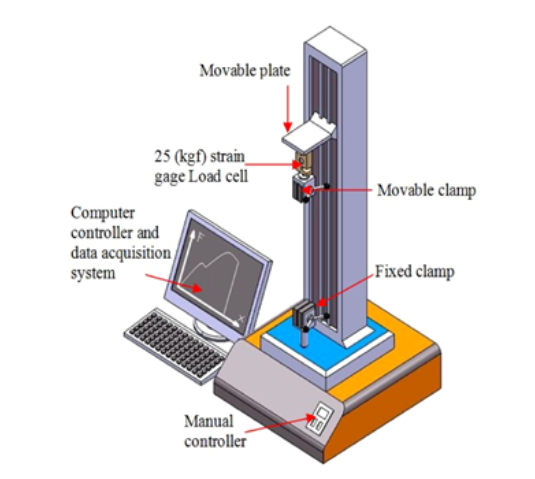

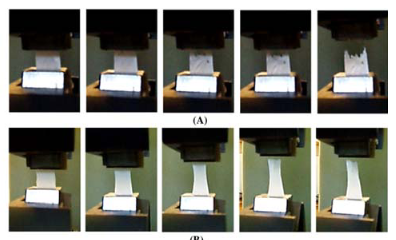


**(a)**

**(b)**

Figure S1. (a) The schematic presentation STM-5 tensile machine illustrated by Taghizadeh et al (29) (b) the process of tensile testing reported by Lee and Deng (30).
